# Gender Representation on Gender-Targeted Television Channels: A Comparison of Female- and Male-Targeted TV Channels in the Netherlands

**DOI:** 10.1007/s11199-016-0727-6

**Published:** 2017-01-05

**Authors:** Serena Daalmans, Mariska Kleemans, Anne Sadza

**Affiliations:** Behavioural Science Institute, Communication Science, Radboud University, Postbus 9104, 6500 HE Nijmegen, The Netherlands

**Keywords:** Gender-targeted channels, Gender stereotyping, Gender representation, Content analysis, Television

## Abstract

The current study investigated the differences in the representation of gender on male- and female-targeted channels with regard to recognition (i.e., the actual presence of men and women) and respect (i.e., the nature of that representation or portrayal). To this end, the presence of men and women on two female- and two male-targeted Dutch channels (*N* = 115 programs, *N* = 1091 persons) were compared via content analysis. The expectation that men’s channels would portray a less equal and more traditional image of gender than women’s channels was generally supported by the results. Regardless of genre as well as country of origin of the program, women were underrepresented on men’s channels, while gender distribution on women’s channels was more equal. The representation of women in terms of age and occupation was more stereotypical on men’s channels than on women’s channels, whereas men were represented in more contra-stereotypical ways (e.g., performing household tasks) on women’s channels. Since television viewing contributes to the learning and maintenance of stereotyped perceptions, the results imply that it is important to strengthen viewers’ defenses against the effects of gender stereotyping when watching gendered television channels, for instance through media literacy programs in schools.

Over the past decades, research has made it abundantly clear that women are underrepresented in the media and that, when they are present, they are more often than not represented in stereotypical roles (Collins 2011; Emons et al. 2010; Furnham and Paltzer 2010; Lauzen et al. 2008; Signorielli and Bacue 1999). Because the roles of women in society have expanded tremendously as a result of the ongoing process of emancipation, these consistent findings are often seen as remarkable (Collins 2011; Emons et al. 2010; Lauzen et al. 2008; Signorielli and Bacue 1999). However, recent developments in the television landscape may provide new insights on the issue. There are indications that specific gender-targeted genres (e.g., soaps and teen scene) might actually showcase both a more equal distribution of men and women as well as less stereotyping in its gender portrayals (Gerding and Signorielli 2014; Lauzen et al. 2006). Following on from this speculation, the emergence of channels that specifically define men or women as their target group and thus predominantly broadcast gender-targeted genres (also called narrowcasting, Kuipers 2012; Smith-Shomade 2004) might be a promising development with regard to a more representative portrayal of both men and women. However, gender portrayal on such gendered channels is rather unexplored, and is therefore central to the current study.

*Narrowcasting,* or organizing user groups into specific audience markets, started from the 1980s onward in the United States (Kuipers 2012; Lotz 2006). It meant that specific, often gendered, audience groups were targeted via specific programming and advertising content (Sheperd 2014). Following this pattern, we term narrowcasting on channels that explicitly target either a female or a male audience “gender-targeted channels” here. Gender-targeted television channels employing narrowcasting currently form an increasingly large portion of the television landscape in a multitude of countries (Kuipers 2012; Smith-Shomade 2004; Van Bauwel 2016). Previous research into narrowcasting has, on the one hand, focused on the emergence of female-targeted channels, such as *Lifetime* and *Oxygen*, and its programming strategies, content choices, and rhetoric used to win female audiences and advertisers of female products (Byars and Meehan 1994; Hundley 2002; Lotz 2006; Meehan and Byars 2000; Tankel and Banks 1997). On the other hand, researchers studied whether specific gender-targeted programs such as *Sex and The City* and *Ally McBeal* can be considered (post-) feminist texts (Akass and McCabe 2004; Dubrofsky 2002). What remains unclear based on previous research is how the gendered focus of such channels and of programs suitable to broadcast on gender-targeted channels affect the representation of men and women in terms of presence and stereotyping.

Investigation of the portrayal of men and women in programs broadcast at gender-targeted channels becomes urgent when considering statements by television scholars who label gender-targeting or narrowcasting as a hegemonic practice (Meehan 1990; Sheperd 2014; Smith-Shomade 2004). Moreover, studying gender representation remains of the utmost importance because watching television still is the most time-consuming pastime (Collins 2011; Signorielli 2012). As a result, television is seen as one of the main institutions associated with disseminating stereotyped views of the world and its gender roles. From the theoretical vantage point of cultivation theory as well as social learning theory, television is confirmed as one of the main agents of socialization (Bandura 1977; Gerbner 1979; Signorielli 2012). Research has shown that television viewing contributes to the maintenance as well as the learning (molding) of gender stereotyped perceptions among children, adolescents, and adults (Larson 2001; McGhee and Frueh 1980; Signorielli 1989; Welch et al. 1979). Furthermore, it is generally accepted that television impacts gender socialization in people’s self-image as well as their image of others (Signorielli 2012). Assuming that television has the potential to shape attitudes, self-perception, and behavior—on top of the idea that stereotyping plays a crucial role in the maintenance of power inequalities within wider social, cultural, political, and economic structures (Cottle 2000; Dyer 1993, 1997; Morgan 2007)—it becomes important to analyze and understand the nature of gender-role portrayals on gender-targeted channels.

Previous research on gender stereotyping in media has generally focused on two levels of gender stereotyping. The first level of gender stereotyping includes the actual presence of men and women in television programs (whether they appear), termed *recognition*. The second level focuses on the nature of that representation or portrayal (how they appear), termed *respect* (Collins 2011; Signorielli and Bacue 1999). Research into narrowcasting remains inconclusive about whether the representation of men and women on gender-targeted channels differs from the overall established patterns of underrepresentation of women (i.e., recognition) and stereotyping portrayal of both men and women (i.e, respect). Moreover, known previous research into narrowcasting has only studied female-targeted channels (Byars and Meehan 1994; Hundley 2002; Lotz 2006; Meehan and Byars 2000; Tankel and Banks 1997), but a comparison between the gender representation on male-targeted and female-targeted channels is lacking. This comparison is important because, if these gender-targeted channels differ in how they represent gender, this might lead to differing conceptions of gender roles and gender aspirations between their male and female target audiences (Gerding and Signorielli 2014).

In all, the question that is central to the current study is: What are the differences in the representation of gender on male- and female-targeted Dutch channels with regard to the characterization concepts of recognition and respect? This question will be investigated by analyzing gender-targeted television channels in the Netherlands, a country in which 40% of the television landscape currently explicitly targets a gendered target audience (Stichting Kijkonderzoek [SKO] 2014).

In their longitudinal analysis of gender on television, Signorielli and Bacue (1999) use presence and gender-role stereotyping as indicators of recognition and respect respectively. These concepts stem from a study of televised racial minorities by Clark (1972), who posited that positive changes in the treatment of lower status groups (minorities including women) can be seen as a process that follows two stages: recognition and respect. In gender research, recognition can be seen as the presence of women on the television screen (Lauzen and Dozier 2005; Signorielli and Bacue 1999). Respect is measured by the extent toward which women are not portrayed in stereotypical roles**,** but rather represented in a diverse manner because this diversity is necessary to be represented fairly and positively (Signorielli and Bacue 1999).

## Recognition: Presence of Men and Women on Television

Recognition is defined as men and women being represented on television proportional to their presence in society (Signorielli and Bacue 1999). Research into gender representation has overwhelmingly found that women are underrepresented on television compared to their presence in society (Collins 2011; Emons et al. 2010; Glascock 2001; Greenberg and Atkin 1980; Koeman et al. 2007; Signorielli and Bacue 1999; Tedesco 1974). The last decades have shown a trend towards a more equal distribution of male and female characters (Elasmar et al. 1999; Emons et al. 2010; Glascock 2001; Greenberg and Collette 1997; Lauzen and Dozier 2005; Vande Berg and Streckfuss 1992), but increases are often small and women remain underrepresented (Koeman et al. 2007; Segijn et al. 2014).

There are some indications that levels of over- or under-representation are connected to the gender of the target audience. Gunter (1986) found that soap operas in 1970s that were specifically geared toward a female audience had an equal distribution of male and female characters. Gerding and Signorielli (2014) similarly found that whereas females were underrepresented in ‘tween programs geared towards male adolescents, the programs geared towards female adolescents mirrored the U.S. population. Based on previous results we posit that women will be underrepresented compared to their presence in society on men’s channels, but not on women’s channels (Hypothesis 1).

Previous studies have revealed that television genres can differ in the ways they represent gender. It has been shown that gender stereotyping is surely still present, but has generally revealed a trend of decreasing stereotyping in genres such as television fiction (Emons et al. 2010; Greenwood and Lippman 2010; Gunter 1986; Signorielli and Bacue 1999), teen scene programming (Gerding and Signorielli 2014), and advertising (Wolin 2003). Moreover, the portrayal of women in these genres has become more representative of the lives and status of contemporary women. In contrast, other studies—mostly on television advertising, but also on fictional programs—conclude that women are still underrepresented and portrayed in a stereotypical way and that the degree of stereotyping is even worsening (Allen and Coltrane 1996; Bretl and Cantor 1988; Ganahl et al. 2003; Harwood and Anderson 2002; Koeman et al. 2007; Milner and Higgs 2004). Based on the latter results, some have argued that, due to its continued gender-stereotyped nature, television forms a lagging social indicator, which reflects “how the economy or society was rather than how it is or how it will be” (Estes 2003, p.4; see also Emons 2011; Kim and Lowry 2005).

Taken together these results reveal that there is inconsistency in the literature about the relation between genre and gender-stereotyping. These possible genre differences become relevant and important when combined with the increased attention from a cultivation perspective that has been given to the possibility of genre-specific cultivation effects (Bilandzic and Busselle 2008; Bilandzic and Rössler 2004; Cohen and Weimann 2000; Grabe and Drew 2007; Morgan and Shanahan 2010). Following the idea put forth by Hawkins and Pingree (1981) that different TV genres may cultivate different views of the world, research has revealed large differences between genres (Gomes and Williams 1990; Koeman et al. 2007; Pennekamp 2011). Because men’s and women’s channels cater to different expected audiences most likely with a selection of different gendered genres, this might influence the gender representation on these channels as a whole. This then leads to the following research question: Do genre-differences play a role in the presence of men and women on gender-targeted television channels? (Research Question 1).

The country of origin of the selected programming might also play a role in gender representational differences between channel types. Previous research has revealed that country of origin plays a significant role in the degree of stereotyping present in the representation of gender (Emons et al. 2010; Furnham and Paltzer 2010; Wiles et al. 1995). For example, Emons et al. (2010) found that U.S. programs on Dutch television represented more male adults, more women involved in childcare, more men involved in a job, and fewer males involved in other activities compared to Dutch programs on the same channels. Based on this comparison, they conclude that American programs on Dutch television are more gender-stereotyped than programs of Dutch origin. Their research indicates that gender portrayals on television can be artifacts of the culture of the society they were created and thereby potentially reflective of the degree of gender equality in the culture of origin. Because the Dutch television landscape hosts a large degree of foreign (especially American) programming, it becomes interesting to see how this affects gender representation on Dutch gendered channels (Kuipers 2008, 2011). We therefore pose the following research question: Do differences in country of origin of the program play a role in the presence of men and women on gender-targeted television channels? (Research Question 2).

## Respect: Stereotyping in Gender Representation

Analyzing the presence of women in the television world is only a relatively small aspect the representation of women on TV. “A more complete understanding of how women are portrayed on television comes from examining the type and depth of the roles in which they are cast – what Clark (1972) referred to as respect” (Signorielli and Bacue 1999, p. 530). We therefore also investigate how women appear in television programs.

The first indicator of respect analyzed in the present study is age. Studies have found that inequalities in the age of televised men and women persist because women are continually represented as younger than their male counterparts are (Emons et al. 2010; Glascock 2001; Signorielli and Bacue 1999). The fact that men on television are predominantly represented as older and therewith are perceived to be wiser than their female counterparts can be interpreted as women being given less respect (Signorielli and Bacue 1999).

Furthermore, previous research on gender-targeted children’s programming has revealed that programs geared toward a boy audience often showcased rather traditional gender-biased portrayals, whereas programs geared toward a girl audience less often featured gender-stereotypical roles and sometimes even showcased counter-stereotypical roles (Banet-Weiser 2004; Gerding and Signorielli 2014; Leaper et al. 2002; Northup and Liebler 2010; Thompson and Zerbinos 1995). Taken together with previous research on age as an indicator of respect (Emons et al. 2010; Glascock 2001; Signorielli and Bacue 1999), we expect a more traditional image on men’s channels than on women’s channels. This leads to the expectations that a larger percentage of women will be young adults on men’s channels than on women’s channels (Hypothesis 2a) and a larger percentage of men will be adults on men’s channels than on women’s channels (Hypothesis 2b).

The second aspect of respect that we will analyze focuses on the social roles in which men and women are cast (Emons et al. 2010; Gerbner 1995; Greenberg and Atkin 1980; Gunter 1986; Signorielli and Bacue 1999). Studies showed that televised men are more likely to be cast in occupational roles, whereas televised women are more likely to be cast in nurturing or marital roles (Gunter 1986; Lauzen et al. 2008; Signorielli and Bacue 1999; Tedesco 1974). Televised women are traditionally represented as housewives who perform housekeeping chores and are preoccupied with family life (Emons et al. 2010; Gunter 1986; Koeman et al. 2007; Signorielli and Bacue 1999). The results on gendered representation of social roles, combined with the previously outlined results regarding the predominance of traditionally gender-stereotyped portrayals in programming geared towards boys and a more gender-balanced representation in programming geared towards girls (Banet-Weiser 2004; Gerding and Signorielli 2014; Leaper et al. 2002; Northup and Liebler 2010; Thompson and Zerbinos 1995), leads us to expect that women on men’s channels will be represented performing household/caregiving tasks more often than women on women’s channels (Hypothesis 3a) and men on men’s channels will be represented performing household/caregiving tasks less often than men on women’s channels (Hypothesis 3b).

In line with this reasoning, studies have found women to be underrepresented with regard to being professionally employed (Elasmar et al. 1999; Gunter 1986; Coltrane and Adams 1997; McNeil 1975; Signorielli 1989; Signorielli and Bacue 1999), which is the next indicator for respect that we will analyze. Over time, there appears to be an increase of women represented as having a job and a decrease of men represented as have a job (Signorielli and Bacue 1999). Again, however, this trend is not straightforward because the occupational status of men and women on television tends to fluctuate (Emons et al. 2010). Nevertheless, due to the expectedly more gender-traditional representation on men’s channels (Banet-Weiser 2004; Gerding and Signorielli 2014; Leaper et al. 2002; Northup and Liebler 2010; Thompson and Zerbinos 1995), we predict that women will be granted less respect on men’s channels in terms of occupational status. Thus we hypothesize that women on men’s channels will be represented as being professionally employed less often than women on women’s channels (Hypothesis 4a) and that men on men’s channels will be represented as being professionally employed more often than men on women’s channels (Hypothesis 4b).

The suggestion that family life and parenthood are of greater significance to women than to men is also implicit in the fact that parental status is more often made explicit for women than for men (Davis 1990; Emons et al. 2010; Glascock 2001; Gunter 1986; McNeil 1975). The final indicator of respect that we will analyze, therefore, is parental status. Again there is a trend toward a more egalitarian distribution of known parental status for characters over time. The percentages of known parental status decreased from 81% for women and 54% for men in McNeil’s (1975) study to 56% for women and 42% for men in Glascock’s (2001) study. Again, a more traditional representation is expected to be more prevalent on men’s channels than on women’s channels (cf. Banet-Weiser 2004; Gerding and Signorielli 2014; Leaper et al. 2002; Northup and Liebler 2010; Thompson and Zerbinos 1995). We therefore expect women on men’s channels to be represented as mothers more often than women on women’s channels will be (Hypothesis 5a) and men on men’s channels to be represented as fathers less often than men on women’s channels will be (Hypothesis 5b).

Combined, these hypotheses test differences in the levels of *recognition* and *respect* given to men and women on men’s and women’s television channels by testing differences in terms of stereotypical or counter-stereotypical role portrayals between the two channel types, comparable to what Gerding and Signorielli (2014) did in their study on ‘tween programs. As we argued, these differences may impact the ideas that men and women have about what are acceptable gender-role patterns and what to expect from oneself and others.

## Method

To test our hypotheses, we conducted a quantitative content analysis of gender-role portrayals of 1091 characters in television 115 programs aired during primetime on four gender-specific television channels in the Netherlands in 2014. All four gendered cable channels on the Dutch television were analyzed: RTL7 and Veronica as men’s channels and RTL8 and Net5 as women’s channels. This distinction was made clear in the explicit statements on their marketing pages as well as their slogans broadcasted during their televised commercial breaks (e.g., “Everything women love,” “What women want,” “RTL7 knows what men want,” and “More for men”). Examples of programs on women’s channels are *15 Kids and Counting* and *Sex and The City*; men’s channels broadcast, for instance, *Top Gear* and programs about soccer. All included channels are commercial broadcasters because Dutch public service broadcasters do not identify specific target audience but are (by law) aimed at informing and entertaining the general population (Koeman et al. 2007).

A check of the validity of the distinction in gendered channels was conducted by verifying the audience profiles, based on gendered viewer ratings, for each channel (Stichting Kijkonderzoek [SKO] 2014, p. 39). The ratings revealed that the audience profile of the male channels RTL 7 and Veronica is indeed male-dominated, with respectively 64.0% and 55.1% of the viewers in 2014 being male. The women’s channels RTL8 and Net5 were shown to have a more female audience. Women represented 64.2% of the audience watching RTL8, and 63.5% of the audience watching Net5. In terms of market share, the male channels RTL7 (5.0%) and Veronica (4.4%) had a combined market share of just below 10%, whereas the women’s channels RTL8 (2.4%) and Net5 (3.5%) had a combined market share of just below 6% (Stichting Kijkonderzoek [SKO] 2014).

### Sample

For each of the four channels, five primetime evenings (6 PM–midnight) of broadcasting were coded and analyzed. The evenings were recorded as part of a larger clustered sample of several constructed weeks and the channels were therefore recorded on consecutive days over the course of several weeks (29 March 2014–12 May 2014). This type of sampling leads to more representative results than recording an actual week (Riffe et al. 2005) because television channels sometimes have thematic weeks that may not be representative of year-long programming. All programs were analyzed but only if the complete episode was aired within the timeframe of the sample. The context unit of our study was one episode, therefore only information that was shown in the specific episode was used for coding.

A total number of 56 programs (48.3%) were broadcasted on a women’s channel: NET5 (26, 22.6%) and RTL8 (30, 26.1%), and 59 programs (51.7%) were broadcasted on a men’s channel: RTL7 (24, 20.9%) and Veronica (35, 30.4%). From the total of 1091 characters that were present in the programs, 597 (54.7%) were presented on men’s channels; 494 (45.3%), on women’s channels.

### Recording Units and Coding Procedure

The two channel types constitute the units of analysis for our study. Recording units were storylines (for fictional programs) and items (for entertainment and reality programs) within programs and the main characters or persons who were featured in them. Entertainment and reality as a genre contained programs, such as *Masterchef* and *Top Gear*, whereas fiction as a genre contained both comedic fiction, such as *Two and a Half Men* and *Mike & Molly,* and dramatic fiction, such as *Criminal Minds* and *The Bold and the Beautiful*. The women’s channels sample consisted of 56 programs, of which 34 (60.7%) were fictional and 22 (39.3%) were entertainment and reality. The men’s channels sample consisted of 59 programs, of which 30 (50.9%) were fictional and 29 (49.1%) were entertainment and reality.

Coding initially differentiated between fictional and non-fictional programming, of which the latter included the genres News and Information and Entertainment and Reality. However, the results revealed that there were actually no programs in the sample which belonged to the News and Information genre.

Based on Emons et al. (2010) up to ten items or storylines, each with up to eight persons, were coded. For fiction these eight characters were selected by coding only main characters in the episode. A main character was defined as a character who plays a leading role in the narrative and whose choices and behavior were essential for the development of the plot (Egri 1960; Lauzen and Dozier 2005; Weijers 2014). In entertainment and reality programs, the eight persons who had the most speaking and/or screen time per item were coded. Show hosts and reporters were coded but excluded from the analyses because they differed from the rest of the population as a result of their function rather than their gender or the channel type on which they were portrayed (i.e., every show host always has an occupation; marital and parental status are very rarely made explicit). Moreover, only a minority of the initial sample consisted of these types of characters (*n* = 50). In addition, animated persons from three program broadcast on men’s channels were also excluded.

The coding instrument used to analyze the persons and characters in the sample (see Table 1 for coding definitions, categories, and frequencies) was developed using prior studies of prime-time television (Davis 1990; Elasmar et al. 1999; Emons et al. 2010; Glascock 2001; Greenberg and Atkin 1980; Koeman et al. 2007; Signorielli and Bacue 1999). Two coders were involved in the coding process. Coders received coder training and independent practice on programs that were not part of the sample. The coders independently coded practice materials and then compared and discussed the results. The coding instrument was edited after these discussions to fix potential problems prior to coding and analysis. After the final revisions in the coding instrument, a little over 10% of the program sample (*n* = 14; 12.1%) was randomly selected to be double coded.

**Table 1:** Overview of coded variables

Variable	Definition	Categories	Kalpha	Frequencies *n* (%)
Genre	Genre to which the program belongs (*N* = 115)	Entertainment and Reality Fiction	1.00	52 (45%) 63 (55%)
Country of origin^a^	Country of origin of the program (*N* = 115)	The Netherlands United States, Great-Britain / Other	1.00	22 *(*20%*)* 85 (73%*)* 4 (3.5%) / 4 (3.5%)
Gender	Person or character’s gender (*N* = 1091)	Male Female	.98	720 (66%) 371 (34%)
Age	Person or character’s age in terms of the life cycle (*N* = 1091)	Child (0–12) Teenager (13–18) Young adult (19–34) Adult (35–49) Middle-aged (50–64) Senior (65+)	.87	21 (1.9%) 45 (4.1%) 408 (37.4%) 455 (41.7%) 116 (10.6%) 46 (4.2%)
Household and caregiving tasks	Whether the person or character engages in household or caregiving tasks (for example: cleaning, doing the laundry, or taking children to school) (*N* = 1091)	Yes No	.93	71 (6.5%) 1020 (93.5%)
Employment	Whether the person or character is portrayed as being professionally employed or explicitly mentions having a job (*N* = 1091)	Yes No Unknown	.96	681 (62.4%) 20 (1.8%*)* 390 (35.7%)
Parental status	Whether the person or character is portrayed as a parent or explicitly mentions being a parent (*N* = 1091)	Yes No Unknown	.96	147 (13.7%) 19 (1.8%) 905 (84.5%).

^a^In the analyses, the categories of “Great-Britain” and “Other” were combined due to low cell frequencies

Intercoder reliabilities were calculated in SPSS using the macro by Hayes for Krippendorff’s alpha (Hayes and Krippendorff 2007). All variables were analyzed as nominal variables except for age, which was analyzed as an ordinal variable. The cut-off points were defined based on Lombard et al. (2002) who suggest that Krippendorff’s alpha coefficients of .90 or higher are always acceptable and .80 or higher are acceptable in most situations. As reported in Table 1, intercoder reliabilities are acceptable for all variables (Kalphas > .87).

## Results

### Presence as an Indicator of Recognition

The first hypothesis stated that women would be underrepresented on men’s channels, but not on women’s channels, compared to their presence in (Dutch) society (Hypothesis 1). In 2014, 49.5% of the Dutch population (*N* = 16,829,289) was male whereas 50.5% of the population was female (Centraal Bureau voor de Statistiek 2014). The gender distribution on men’s and women’s channels was examined with a Chi square goodness of fit test whereby the societal percentage was used as the expected value. The results showed that women were significantly underrepresented on men’s channels compared to their presence in society. Women made up only 22% of the cast, whereas men (78%) were much more present compared to the gender distribution in society, χ^2^(1, *n* = 597) = 192.48, *p* < .001, *Cramer’s V* = .57. In contrast, women (and men) on women’s channels were not underrepresented. Their presence did not differ significantly from society in terms of gender. Women composed 48.6% of the population whereas men composed 51.4%, χ^2^(1, *n* = 494) = .73, *p* = .394, *Cramer’s V* = .04. In all, the results provide support for Hypothesis 1 because underrepresentation of women was only found on men’s channels.

The first research question asked if the genre of the programs presented on men’s and women’s channels would lead to a differing presence of men and women on these channels. The results revealed that, based on the adjusted residuals on men’s channels, the percentage of men significantly exceeded expected frequencies within the entertainment and reality genre (87.9%, adjusted residuals =8.1), whereas the percentages for women (although still underrepresented) significantly exceeded expected frequencies within the fictional genre on men’s channels (40.1%, adjusted residuals =8.1), χ^2^(1, *n* = 597) = 65.20, *p* < .001, *Cramer’s V* = .33. In contrast, on women’s channels the percentage of women significantly exceeded expected frequencies within the entertainment and reality genre (54.8%, adjusted residuals =2.4), whereas the percentage of men (55.9%, adjusted residual*s* = 2.4) significantly exceeded expected frequencies in fictional genre, χ^2^(1, *n* = 494) = 5.57, *p* = .018, *Cramer’s V* = .11. In all, there is a more pronounced difference in the representation of gender on men’s channels in different genres than on women’s channels, where gender is more evenly divided.

The second research question asked if country of origin of the programs presented on men’s and women’s channels would lead to a differing presence of men and women on these channels. Overall, results showed that women were underrepresented in programming from all countries. However, on men’s channels in programs created in the United States, the percentage of women significantly exceeded expected frequencies (34%, adjusted residuals =8.0) with the percentage of men (66.0%) falling below expected rates, χ^2^(2, *n* = 597) = 64.85, *p* < .001, *Cramer’s V* = .33. Comparatively, men are starkly overrepresented in programming from both the Netherlands (95.2%, adjusted residuals = 5.8) and Other countries (91.1%, adjusted residuals =3.7), with the percentage of women falling below expected rates (NL: 4.8%, Other: 8.9%). In contrast, on women’s channels there were no significant differences between countries of origin in the presence of men and women, χ^2^(2, *n* = 494) = 4.35, *p* = .114, *Cramer’s V* = .10.

These results prompted a closer inspection of the particular programming originating in the Netherlands and Other countries in the sample. Programs on men’s channels that were created in The Netherlands were predominantly sports talk shows (*n* = 4) and reality crime shows (*n* = 5). We need to note here that Dutch programs constitute only 17.9% of the programming on men’s channels. All other programs were originally from United States (71.4%) or from Great Britain and other countries (10.7%). Programs on men’s channels from other countries were all male-oriented shows such as *Top Gear*, the famous *BBC* car talk show. Comparatively, the programs aired on men’s channels originating in the United States are a little more mixed, consisting of for example the reality crime show *Cops* as well as comedy shows such as *Mike & Molly* and drama such as *Criminal Minds*.

### Age as Indicator of Respect

The second set of hypotheses stated that women would be represented as young adults on men’s channels more often than on women’s channels (Hypothesis 2a), whereas men would be portrayed as adults on men’s channels more often than on women’s channels (Hypothesis 2b). The distribution of women along age categories differed significantly between channel types, χ^2^ (5, *n* = 372) = 13.70, *p* = .018, *Cramer’s V* = .19 (see Table 2a). In accordance with hypothesis 2a, the residual analysis revealed that on men’s channels over half of all women (56.8%, adjusted residuals =2.7) were portrayed as young adults which was significantly more than the 42.1% on women’s channels*.* As predicted in Hypothesis 2b, 49.2% of men (adjusted residuals *=*2.9) were represented as adults on men’s channels, and this was also significantly more than the 37.8% on women’s channels, χ^2^(5, *n* = 719) = 19.04, *p* = .002, *Cramer’s V* = .16. Although not hypothesized, these results also revealed that adult women (40.0%, adjusted residuals *=*2.8) were significantly more present on women’s channels than on men’s channels (25.8%).

**Table 2:** Indicators of respect for women and men on gender-targeted channels

	Represented women		Represented men	
Indicators	Men’s channels	Women’s channels	Men’s channels	Women’s channels
(a) Age				
Child	3.8%	1.7%	.4%	3.9%^*^
Teenager	4.5%	4.2%	3.7%	4.7%
Young adult	56.8%^*^	42.1%	31.0%	34.6%
Adult	25.8%	40.0%^*^	49.2%^*^	37.8%
Middle aged	3.0%	7.5%	12.3%	14.6%
Senior	6.1%	4.6%	3.4%	4.3%
* n*	132	240	465	254
(b) Household and caregiving tasks				
Yes	12.1%	11.7%	1.5%	7.9%^*^
No	87.9%	88.3%	98.5%^*^	92.1%
* n*	132	240	465	254
(c) Employment				
Yes	43.2%	57.1%^*^	64.9%	72.8%^*^
No	3.0%	4.6%	.4%	1.2%
Unknown	53.8%^*^	38.3%	34.3%^*^	26.0%
* n*	132	240	465	254
(d) Parental status				
Yes	19.7%	23.8%	5.4%	15.4%^*^
No	.8%	5.4%^*^	.2%	1.6%^*^
Unknown	79.5%	70.8%	94.4%^*^	83.1%
* n*	132	240	465	254

^*^Frequency significantly exceeded expectations by adjusted standardized residuals

As the percentages above already indicate, most of main characters in the sample were either young adults or adults. The other age categories that we discerned (child, teenager, middle-aged adults, and seniors) appeared less frequent (12.3–14.6% for middle aged men, all other percentages <7.5%, see Table 2a). No notable differences in presence of men and women of these age categories on either men’s or women’s channels were found, except for significantly more male children on women’s channels (3.9%, adjusted residuals *=* 3.5) than on men’s channels (.4%).

### Tasks as Indicators of Respect

We expected that women on men’s channels would be overrepresented performing household or caregiving tasks compared to women on women’s channels (H3a). We did not find support for this hypothesis, since 12.1% of women on men’s channels and 11.7% of women on women’s channels performed these tasks and these numbers did not differ significantly, χ^2^(1, *n* = 372) = .017, *Cramer’s V* = .007, *p* = .897 (see Table 2b).

It was further hypothesized that men on men’s channels would be significantly underrepresented performing household or caregiving tasks compared to men on women’s channels (Hypothesis 3b). The results supported this hypothesis as 7.9% of men on women’s channels performed household or caregiving tasks (adjusted residuals *=*4.3) compared to only 1.5% of men on men’s channels, χ^2^(1, *n* = 719) = 18.44, *p* < .001, *Cramer’s V* = .16 (see Table 2b).

### Employment as Indicator of Respect

Women on men’s channels were expected to be represented as having an occupation less often than women on women’s channels (Hypothesis 4a), whereas men on men’s channels were expected to be represented as having an occupation more often than men on women’s channels (Hypothesis 4b). A significantly larger percentage of women (57.1%, adjusted residuals *=* 2.6) was indeed portrayed as having an occupation on women’s channels than on men’s channels (43.2%), χ^2^ (2, *n* = 372) = 8.31, *p* = .016, *Cramer’s V* = .15 (see Table 2c). This finding confirms Hypothesis 4a. Contrary to Hypothesis 4b, men on men’s channels were portrayed as having an occupation significantly less often than men on women’s channels were (see Table 2c). On men’s channels, 64.9% (adjusted residuals *= −* 2.6) of the men were portrayed as having an occupation compared to 72.8% of men on women’s channels, χ^2^(2, *n* = 719) = 6.75, *p* = .035, *Cramer’s V* = .10. Furthermore, the results also revealed a significant overrepresentation within the unknown category of occupation for both women (53.8%, adjusted residuals *=* 2.9) and men (34.6%, adjusted residuals *=* 2.4) on men’s channels.

### Parental Status as Indicator of Respect

The final set of hypotheses predicted that women on men’s channels would be represented as mothers more often than women on women’s channels (Hypothesis 5a) and, conversely, that men on men’s channels would be represented as fathers less often than men on women’s channels (Hypothesis 5b). The results revealed that women’s parental state significantly differed between the channel types, χ^2^(2, *n* = 372) = 6.41, *p* = .040, *Cramer’s V* = .13 (see Table 2d). However, the residual analysis shows that this difference is due to the knowledge of explicit childlessness of women between channel types, where women were significantly more often explicitly childless on women’s channels (5.4%, adjusted residuals *=* 2.3) than on men’s channels (.8%). There was no significant difference between the representation of women as mother between men’s channels (19.7%) and women’s channels (23.8%). We thus need to reject Hypothesis 5a. The results also indicated that men were, as expected in Hypothesis 5b, significantly more often represented as fathers (15.4%, adjusted residuals *=* 4.5) on women’s channels than on men’s channels (5.4%), χ^2^(2, *n* = 719) = 25.08, *p* < .001, *Cramer’s V* = .19 (see Table 2d). Hypothesis 5b is thus supported.

## Discussion

The primary goal of our study was to provide insight into the way that gender was represented on television channels targeting either men or women. The overarching expectation that, due to the target audience, men’s channels would portray a less equal and more traditional image of gender than women’s channels would in terms of both recognition and respect was generally supported by our results. In line with the results of Gerding and Signorielli (2014), our study revealed that whereas women were grossly underrepresented on men’s channels, gender distribution on women’s channels mirrored the Dutch population. This phenomenon cannot be explained by the idea that audiences would prefer watching members of their own gender because women should then have been overrepresented on women’s channels just like men are on men’s channels. Because this was not the case, we can conclude that in contrast to women’s channels, men’s channels show a lack of recognition for women. Moreover, an exploration of the level of recognition per genre and per country of origin of the programs on the gendered channels also revealed that women’s channels, regardless of genre as well as the country of origin of the program, showcased a more equal presence of men and women than men’s channels did. In all, this means that only women’s channels fulfill the first of the two stages towards positive and fair treatment of women: recognition (Clark 1972; Lauzen and Dozier 2005; Signorielli and Bacue 1999).

In addition, our results revealed that men’s channels also lagged behind in terms of the second stage of positive and equal portrayal (Clark 1972): respect. First, the stereotypical value of youth for women (as an indicator of a lack of respect, see also Davis 1990; Emons et al. 2010; Lauzen and Dozier 2005; Signorielli and Bacue 1999) is much more pronounced on men’s channels than it is on women’s channels. Women on women’s channels were not only represented as older than they were on men’s channels, but they were also distributed more evenly over the age categories of young adult and adult. Second, in terms of occupational status, women on women’s channels were more likely to be portrayed as being professionally employed than women on men’s channels were.

However, the results for the other indicators of respect revealed a less straightforward conclusion. As for the percentage of women performing household or caregiving tasks and the overrepresentation of women as parents, there were no significant differences between men’s and women’s channels. However, it should be noted that women’s channels were more likely than men’s channels were to represent men as performing household and caregiving tasks and being parents. Household and caregiving tasks especially were divided rather equally between both genders—in contrast to the results by Emons et al. (2010). Therefore we conclude that at least the degree of gender stereotyping (at least in terms of these indicators) is lower on women’s channels and that they thereby still grant more respect to women and men than men’s channels do.

The most striking result found in our study is that it is the representation of men (rather than women) in which the true differences between men’s and women’s channels are found. Men are represented in counter-stereotypical ways on women’s channels and become connected to the home and family almost as much as women. This focus on home and family life on women’s channels could be interpreted as a post-feminist resurgence of the focus on gender differences and stereotypical feminine values on women’s channels (Gill 2007; McRobbie 2009). Another, more positive, interpretation might, however, be that the private domain is being re-evaluated. Women’s channels seem to present an image in which home and family life are important—for both men and women. This might be interpreted as the private domain itself increasingly gaining respect on these channels. Also, besides their connection to family life, high percentages of both men and women are represented as being professionally employed on these channels, resulting in a more diverse depiction of both genders.

### Limitations and Directions for Future Research

Although the findings here are of clear importance in adding to our understandings of the differences in gender representation on gender-targeted channels, there are some limitations to our study that should be addressed. First, the sample consisted of a constructed week recorded on consecutive days. Therewith, we did not control for seasonal differences in programming. Recording several weeks, one in each season may contribute to the representativeness of the results.

Second, in terms of variables we decided to code as many of the aspects that were associated with the central concepts of recognition and respect as discerned by Clark (1972) and Signorielli and Bacue (1999). To this end, we closely followed previous codebooks (Davis 1990; Elasmar et al. 1999; Emons et al. 2010; Glascock 2001; Greenberg and Atkin 1980; Koeman et al. 2007; Signorielli and Bacue 1999) in the operationalization of our variables. As a consequence, we were not able to explore every one of the aspects in an in-depth manner. For example, household and caregiving tasks were coded as either being performed or not performed. Because of this, if one character was portrayed performing household tasks for an entire episode and another performed only one such task, both were coded as performing these tasks in the same way. It is recommended that future research would measure the number of tasks performed by each character so as to gain more insight into gendered task distribution.

Furthermore, with regard to the variable of occupation, the coding based on previous work relatively simplistically reflected if a person had a job or not (or if it was unknown). Even though the differentiation between having a job or not captures implicit messages of gendered worth in the public sphere, future research should add a dimension of gender-role stereotyping to this variable in building on previous work (Coltrane and Adams 1997; Glascock 2001; Signorielli 1989; Signorielli and Bacue 1999; Signorielli and Kahlenberg 2001). Future research could establish for gendered channels if there are differences between men and women with regard to the type of occupation, the prestige associated with that occupation, as well as the authority they have over other workers in that occupation in order to determine how stereotypical the representation of work for men and women is on these channels. This is important because media portrayals provide one of the sources of information against which people in modern societies give meaning to in their work and family lives (Coltrane and Adams 1997; Signorielli and Kahlenberg 2001). Seen in this light, the media’s previously documented tendency of linking men with jobs in which they have authority and women with jobs with less prestige and less authority over other workers (Coltrane and Adams 1997; Glascock 2001; Signorielli 1989), potentially aids in the perpetuation of gender stereotypes and the maintenance of the gendered status quo.

Third, we decided to exclude show hosts and reporters in entertainment and reality programs because there was almost no variation with regard to the dependent variables. Although their presence was low compared to the other characters who were coded, their representation is important against the background of the current study. For instance, talk shows are frequently watched, and the position of host carries a certain amount of authority and expertise with it, implying that the gender representation of show hosts may also contribute to viewers’ stereotyped views of the world and its gender roles. Previous research has already outlined that men form the majority of show hosts in programs devoted to “hard” content (e.g., politics and economy), whereas women are dominant as show hosts for “soft” topics (e.g., family and romance) (Gerbner 1995; Koeman et al. 2007; Segijn et al. 2014). Therefore, future research should include an in-depth analysis of these roles on gendered channels to assess potential differences.

In addition to including show hosts and reporters in future analyses, it might be interesting to explore the relationship of the gender of individuals behind the scenes of the gender-targeted television channels (e.g., managers, writers, directors, advertisers) and the on-screen portrayal of men and women. This is particularly interesting because it could explore the assumption which some media analysts and scholars have put forward that if more women had positions of authority behind the scenes in the entertainment world, the media would offer less gender-stereotyped portrayals of men and women (Benét 1978; Lauzen and Dozier 1999; Mills 1988). Some of these dynamics have been explored for prime-time programming by other researchers (e.g., Glascock 2001; Lauzen and Dozier 1999; Lauzen et al. 2006; Lauzen et al. 2008), but never systematically for gendered television channels.

A final recommendation for future research would be to complete a cultivation analysis to measure how the audiences’ conceptions of gender are affected by the programming on these gendered channels (Gerbner et al. 1978; Morgan 2007; Signorielli 1989; Signorielli and Bacue 1999). This directive for future cultivation research is further strengthened by research based in social role theory (Eagly and Wood 2012) which proposed that gender stereotypes might change when gender distribution changes (Diekman and Eagly 2000), and it is encountered in daily life or through mediated exposure (Eagly and Steffen 1984). Future research could then assess if more traditional or progressive role-pattern expectancies are indeed cultivated in the audiences of men’s and women’s channels respectively. This would necessitate surveying viewers of both types of channels with varying levels of exposure to television about their conceptions of gender roles. As outlined previously, some of the findings presented in our study are concerning; however, until a cultivation analysis is conducted, the actual influence of these programs on actual perceptions of gender and gender roles remain unknown.

### Practice Implications

Television broadcasters work fervently to entertain and keep their audiences watching and loyal, all in order to turn a profit by selling advertising space to makers of products and services aimed at specific (gendered) niche audience groups (Kuipers 2012; Turow 1998). However, through their politics of narrowcasting, viewers—and particularly viewers of men’s channels our study points out—are at risk for developing too narrow conceptions about gender roles that may prove to be limiting in real life. Because research has shown that television viewing contributes to the maintenance, as well as the learning, of gender stereotyped perceptions (Gerbner et al. 1978; Larson 2001; McGhee and Frueh 1980; Welch et al. 1979), we would therefore argue that the everyday television viewer should be made aware of the presence of stereotyping on television. A possible way to do this is to pay attention to this issue in media literacy programs, which are part of school curricula in an increasing number of schools (Koltay 2011; Tuominen and Kotilainen 2012). In these lessons, educators at all levels of education should sensitize their students about gender-role depictions in television programming and the (possible) effects they have on men and women. In this way, they would ideally be providing a continuous strengthening of children’s, adolescents, and young adults’ defenses against the effects of gender stereotyping they may encounter when watching various (gendered) television channels throughout their life.

### Conclusion

To conclude, some context for the rise of men’s and women’s channels might be provided by the idea that we now live in a post-feminist era in which there has been a resurgence of the belief in sexual difference (Gill 2007; McRobbie 2009). It has also been suggested that the increased status of women in society has put pressure on the concept of masculinity (Beynon 2002), which could explain the more traditional gender portrayal on channels aimed at male audiences. However, whatever the cause of the matter may be, our study makes clear that rather than mirroring emancipatory changes in society via a trend towards a more equal representation of gender on television, the Dutch television landscape has become divided through some large differences in the way gender is represented on men’s versus women’s channels.

Taken together, we can conclude that, in the Netherlands, it seems that a more equal image of gender might be cultivated particularly for female audiences, while the messages cultivated for male audiences remain highly gender-stereotyped. These findings highlight a worrisome phenomenon when combined with several complexities in gender-related practices and attitudes in the Netherlands. The Netherlands is generally regarded as one of the more gender-equal countries in the world (United Nations Development Program [UNDP], 2015), due for example to the continuously growing working force participation of women, the increasing percentage of women with a college degree, the increase of women with a seat in parliament, a growing participation of men in household chores as well as childcare in the last decades, and a continuous increase in the share of individuals who do not favor gender stereotypes in upbringing, education, and the workplace (Arends-Tóth and van de Vijver 2007; Collier et al. 2013; Emons 2011; Gesthuizen et al. 2002; UNDP 2015). However, the Netherlands also recently dropped three places in the Gender Gap Index (World Economic Forum 2016) due to the fact that in the Netherlands women’s workforce participation rate is still lower than men’s. Women generally have part-time jobs, earn considerably less than men do, and are severely underrepresented in senior executive positions. Considering these complex and conflicting realities in gender-related practices and attitudes in the Netherlands, we feel that the results of our study seen in the light of cultivation theory (Gerbner et al. 1978) and social learning theory (Bandura 1977), combined with the persistent finding that men in general tend to hold more traditional gender views than women do (Bolzendahl and Myers 2004; Brewster and Padavic 2000; Cameron and Lalonde 2001), highlight issues that are important and relevant to consider when examining the potential effects of these gendered representations. As such, the gendered representation on particularly men’s TV channels might form a roadblock that stands in the way of true emancipation and ideas of gender equality being reinforced in the minds of not only women but particularly men.
